# A Proposal of a Personalized Surveillance Strategy for Gastric Cancer: A Retrospective Analysis of 9191 Patients

**DOI:** 10.1155/2019/3248727

**Published:** 2019-01-22

**Authors:** Si-wei Pan, Peng-liang Wang, Han-wei Huang, Lei Luo, Xin Wang, Tao Wang, Fu-nan Liu, Hui-mian Xu

**Affiliations:** Department of Surgical Oncology, The First Affiliated Hospital of China Medical University, Shenyang 110001, China

## Abstract

**Background:**

In gastric cancer, various surveillance strategies are suggested in international guidelines. The current study is intended to evaluate the current strategies and provide more personalized proposals for personalized cancer medicine.

**Materials and Methods:**

In the aggregate, 9191 patients with gastric cancer after gastrectomy from 1998 to 2009 were selected from the Surveillance, Epidemiology, and End Results database. Disease-specific survival was analyzed by Kaplan-Meier method and the log-rank test. Cox proportional hazards regression analyses were used to confirm the independent prognostic factors. As well, hazard ratio (HR) curves were used to compare the risk of death over time. Conditional survival (CS) was applied to dynamically assess the prognosis after each follow-up.

**Results:**

Comparisons from HR curves on different stages showed that earlier stages had distinctly lower HR than advanced stages. The curve of stage IIA was flat and more likely the same as that of stage I while that of stage IIB is like that of stage III with an obvious peak. After estimating CS at intervals of three months, six months, and 12 months in different periods, stages I and IIA had high levels of CS all along, while there were visible differences among CS levels of stages IIB and III.

**Conclusions:**

The frequency of follow-up for early stages, like stages I and IIA, could be every six months or longer in the first three years and annually thereafter. And those with unfavorable conditions, such as stages IIB and III, could be followed up much more frequently and sufficiently than usual.

## 1. Introduction

Despite the incidence and mortality of gastric cancer decline worldwide recently, it is still the fifth most common malignancy and the third cancer-related death disease [1, 2].

As a vital and widely accepted tumor outcome, tumor relapse rates of gastric cancer patients after radical resection vary from 1.6% to 42% for different stages [3–6]. For early detection of relapse, surveillance is one of the most important methods and is also recommended in gastric cancer treatment guidelines [7–10]. Surveillance could also provide some critical information, such as the treatment response as well as the complication status after certain therapy and the nutrition status of patients. Additionally, it also played an important role in detecting the metachronous tumor and giving psychological support [11].

However, there is still no high-level evidence for the best surveillance strategy [11–15]. According to the directions in different guidelines, there are two major categories: the intensive surveillance strategy under a nonsymptomatic situation, represented by the National Comprehensive Cancer Network (NCCN) and Japanese Gastric Cancer Association (JGCA) treatment guidelines, and the symptom-driven follow-up strategy, represented by the National Institute for Health and Care Excellence (NICE) and European Society for Medical Oncology (ESMO) [7–10]. Comparisons from previous studies showed that intensive surveillance is superior to the latter by comparing the survival indexes of patients with symptomatic or asymptomatic recurrence [16, 17]. In contrast, Peixoto et al. [18] indicated that there was no significant difference on overall survival (OS) between two categories. By the way, a previous study indicated that intensive surveillance was verified to be better than the other scheme of prognosis of colorectal cancer [19].

In the NCCN guideline, it suggests that the follow-up should be performed every 3-6 months for the first two years after resection, then every 6-12 months until the fifth year, and annually thereafter [7]. The shortcoming is lack of specificity, such as no distinctions among different substages. Although the surveillance strategy distinguished stage I from stages II-III in the JGCA guideline, it is still not personalized enough [8]. As well, from the perspective of the health economic, being frequently followed up may produce unnecessary cost and increase social pressure, especially for those under the early stage. Part of patients with the early stage could also relieve unnecessary psychological burden and improve quality of life through being followed up not so close as other patients with unfavorable conditions [20, 21].

Conditional survival (CS), considering the changing hazard rate with increased survival time, is one of the prognostic indexes that could dynamically provide more exact assessment on the prognosis. Previous studies indicated that the amplitude of CS was smaller in the patients with favorable clinicopathological factors and larger in those under unfavorable conditions [22–25]. And in colon analysis, patients with lower CS or unfavorable conditions were suggested to be followed up more intensively [26].

Consequently, the intent of this study was to evaluate the surveillance strategies mainly directed in the NCCN guideline, carried out presently by CS and patients from the Surveillance, Epidemiology, and End Results (SEER) database. We supposed that following up of stage I and IIA patients every six months or longer in the first two years is a better solution. And those with higher stages could be followed up every three months or more frequently.

## 2. Material and Methods

### 2.1. Patient Source

The SEER program is a public and annually updated database. It has published information on the incidence and survival data of various cancers and covers approximately 26% of the US population [27]. The population of the current study was selected from the SEER database, and we had obtained permission to access research data files with the reference number 15582-Nov 2016. Moreover, the data did not include the use of human subjects or personal identifying information.

The cohort of the current study included patients who underwent gastrectomy and diagnosed with gastric adenocarcinoma (International Classification of Disease for Oncology, third edition (ICD-O-3) code in the range of 8000–8152, 8154–8231, 8243–8245, 8250–8576, 8940–8950, and 8980–8990) between 1998 and 2009. Patients with stage I to stage III were included in our study based on the seventh edition of the American Joint Committee on Cancer (AJCC) staging manual [28], stage IV patients were excluded for extremely poor prognosis, and the evaluation of surveillance strategy for these patients may be meaningless. Patients who died of causes unknown or other diseases may influence our results. Therefore, only patients who died of gastric cancer were included for analyses. In addition, patients were excluded according to the following criteria: (1) less than 18 years old or older than 90, (2) the clinicopathological or follow-up information was unfaithful or not otherwise specified (NOS), and (3) the survival time was less than one month. Moreover, patients whose tumor is located at the cardia or esophagogastric junction (site code C16.0) were also excluded from the study according to the seventh edition of the AJCC staging manual [28]. Finally, 9191 patients were selected for our further analyses.

The following demographic and pathological characteristics were selected for analyses: race, sex, age at diagnosis, grade, size and the primary site of tumor, extent of the invasion, number of examined and positive lymph nodes (LNs), follow-up time, and survival data at the last time of follow-up (Nov. 2016). The TNM stage was classified by the seventh edition of the AJCC staging manual [28].

### 2.2. Statistical Analysis

The continuous variables of the cohort are presented as mean ± standard deviation (SD), and the categorical variables are described as counts and proportions. Disease-specific survival (DSS) was calculated using the Kaplan-Meier method, and the comparison was identified by the log-rank test. Univariate and multivariate Cox proportional hazards regression models were applied to identify the independent prognostic factors among potential clinicopathological characteristics.

**Figure 1:**
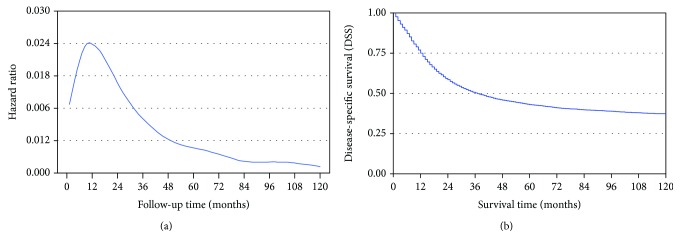
(a) Hazard ratio of death from gastric cancer and (b) actuarial disease-specific survival (Kaplan-Meier survival curve) for 9191 patients are illustrated, and the former showed a high risk of death in the first two years after resection.

The risk of death after gastrectomy is not constant all the while, and the highest risk point is located in the first two years after resection (Figure 1(a)) [22, 23]. Thus, the survival probability of patients who have survived for two years may be changed. Furthermore, patients were grouped according to different TNM stages and the hazard ratio (HR) curves of each stage were used to calculate maximum HR (maxHR) and the corresponding time.

Considering this dynamic changing, we used CS to evaluate the survival at the certain time point. The mathematical formula for CS can be showed as follows: CS(*y* | *x*) = *S*(*x* + *y*)/*S*(*x*), where *S*(*x*) represents the actual survival rate (*S*) at the time point of *x* and *y* represents the additional expected survival time after the time point of *x* [22, 25]. This can be illustrated that if someone has survived one year after resection, then his probability of living an additional year is estimated as CS(1*y* | 1*y*) = *S*(2*y*)/S(1*y*). In the current study, we simulated surveillance strategy by estimating CS after each follow-up at different intervals. Such as the NCCN guideline directing in the first two years after resection, CS is estimated using CS(*x* + 3 *m* | *x*) = *S*(*x* + 3 *m*)/*S*(*x*) after each follow-up and at intervals of three months in order to evaluate the probability of surviving at the next follow-up.

The analyses were carried out by the R software (version 3.4.3; R Foundation for Statistical Computing, Vienna, Austria) and SPSS (version 23.0; SPSS Inc., Chicago, IL), and two-tailed *P*value < 0.05 was considered to be statistically significant in all analyses.

## 3. Results

### 3.1. Clinicopathological Characteristics

As a whole, 9191 patients from the SEER database who underwent gastrectomy and conformed to the screening conditions of the study were included. The demographic and pathological characteristics of the included patients were illustrated in Table 1.

### 3.2. Actual Disease-Specific Survival (DSS)

The five-year DSS was 37.8% in the whole cohort, and at the last time of follow-up, a total of 5599 (60.9%) patients died of gastric cancer (Figure 1(b)). Univariate and multivariate Cox regression analyses were designed to identify the independent prognostic factors associated with DSS (Table 2). In the univariate analysis, age, grade, tumor size, TNM stage, and LNs examined were identified as significant prognostic factors (all *P* < 0.05). Further multivariate analysis confirmed that age, TNM stage, and LNs examined were independent prognostic factors (all *P* < 0.05).

### 3.3. Conditional Survival (CS) and Hazard Ratio (HR) Curves

**Figure 2:**
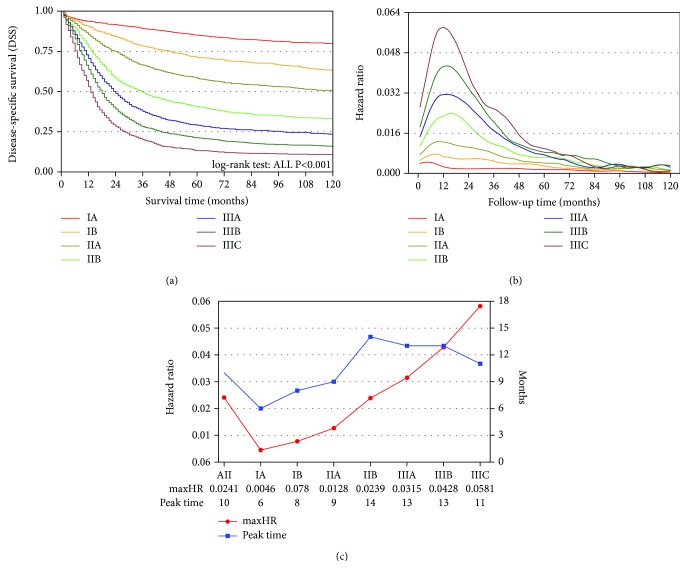
9191 patients were distinguished into seven groups according to the seventh AJCC staging manual from IA to IIIC: (a) survival curves of seven groups and significant differences on survival were demonstrated. (b) HR curves of seven groups were demonstrated, and the curves of stages I and IIA were flatter than those of stages IIB and III, while those of stages IIB and III had an obvious peak in each curve. (c) The maxHR and the corresponding time were also estimated in each group, and it showed a monotonic increasing of the maxHR from stage IA to IIIC.

As indicated in the HR curve, the risk of death was not constant all the while and the highest risk point was in the first two years after resection (Figure 1(a)). In Figure 2(a), it showed significant heterogeneity on survival among TNM stages (all *P* < 0.05). And the risk of death was evaluated over time by HR curve and compared with each other [7, 8]. As illustrated in Figure 2(b), several conclusions were drawn as follows: (1) the curves of stage I patients were flat with no visible change, (2) although stage IIA has a peak, it was relatively flat as a whole, while stage IIB had a visible peak and was more similar to stage III, and (3) the monotonic increasing of HR from stage IA to stage IIIC was intuitively demonstrated in Figure 2(c), especially in the first two years after resection. An example of the third conclusion above involved that the maxHR was 0.0581 and the peak was at 11 months after resection for stage IIIC, while stage IIB was only 0.0239 and at 14 months (Figure 2(c)).

### 3.4. Evaluation of the Surveillance Strategy Using CS

**Figure 3:**
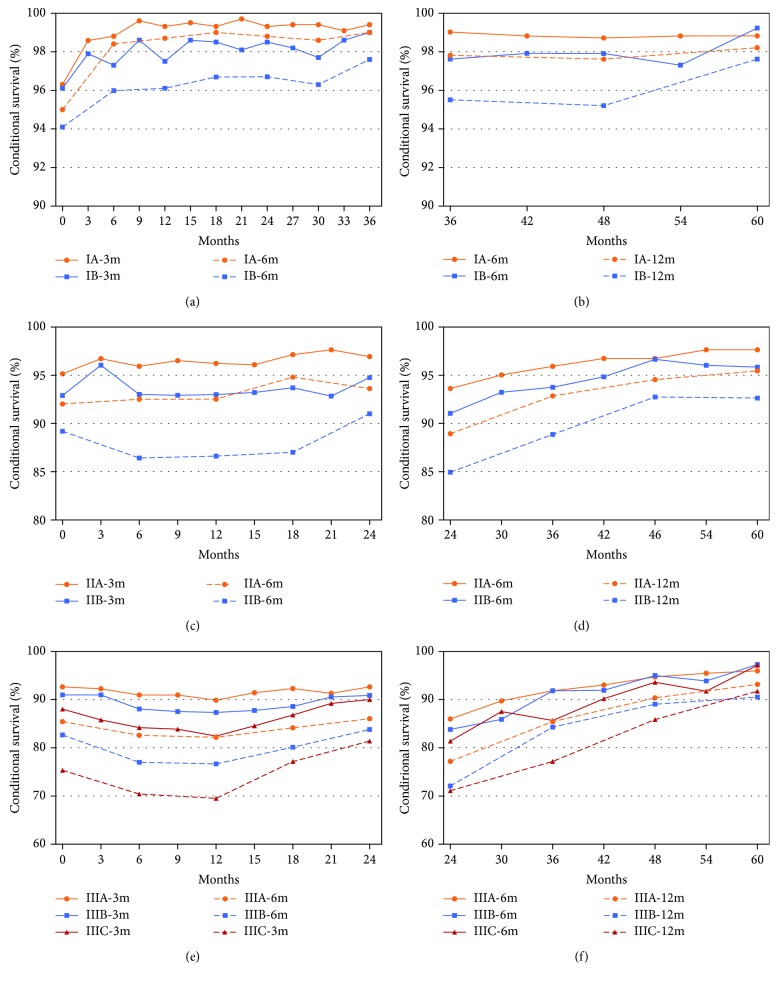
Conditional survivals from different intervals are compared for patients in the cohort differed from TNM stages during different periods: (a) three-month conditional survival (3mCS) and six-month conditional survival (6mCS) of stage I in the first 3 years after resection and (b) 6mCS and 12-month conditional survival (12mCS) in the latter 2 years were demonstrated, and all were at a high level of more than 94%; (c) 3mCS and 6mCS of stage II in the first 2 years and (d) 6mCS and 12mCS in the latter 3 years were demonstrated, and it showed a smaller gap of each pair in stage IIA than stage IIB; (e) 3mCS and 6mCS of stage III in the first 2 years and (f) 6mCS and 12mCS in the latter 3 years were demonstrated. Short intervals of follow-up showed significant superiority of CS in advance stages.

As illustrated in Figure 3(a), three-month CS (3mCS) and six-month CS (6mCS) were both higher in stage IA patients who had already survived 0 to 3 years after resection than stage IB patients but were all kept at a high level of more than 94%. The first three-year follow-up strategy for stage I patients was based on the JGCA guideline differing from the NCCN [7, 8]. The same conclusion could be drawn from 6mCS and 12-month CS (12mCS) from the fourth to the fifth year after resection (Figure 3(b)). In stage II, CS of stage IIA was kept at a high level of more than 90% in the first two years, while 6mCS of stage IIB was lower than 90% differing obviously from 3mCS (Figure 3(c)). And there was a similar situation in 6mCS and 12mCS from the third to the fifth year (Figure 3(d)). Otherwise, the disparities between 3mCS and 6mCS of the first two years were illustrated distinctly in Figure 3(e) among stages IIIA, IIIB, and IIIC. Similar differences were also illustrated between 6mCS and 12mCS in the latter three years (Figure 3(f)). And from these, we concluded that short intervals of follow-up showed significant superiority of CS in advanced stages, including stages IIB and III.

## 4. Discussion

During our clinical work, we found that short interval of follow-up was not necessary for part of gastric cancer patients with an early stage and would increase the economic and psychological burden, while being followed up frequently could find problems timely for advanced-stage patients. According to these, the current study was designed to evaluate the surveillance strategies carried out presently and propose a more individualized improvement. We used HR curve and CS to evaluate the follow-up and finally concluded that patients with an early stage could not be followed up so close and an advanced stage could be more frequently.

As directed in different guidelines, the surveillance after gastrectomy of gastric cancer is necessary for detecting relapse and metachronous tumor and can also supervise the complication after resection, psychological support, and data collection for research [12]. But there is no unified strategy that can be the best choice in all situations [11–15]. In the NCCN guideline, the follow-up should be done at intervals of 3 to 6 months, 6 to 12 months, or annually in different periods [7]. JGCA directs differently from stage I to stages II-III, in which stage I patients should be followed up every six months for the first three years after resection and annually thereafter for two years, while stages II-III should be done similarly to that of the NCCN guideline [8]. ESMO and NICE are only suggested to be followed up when symptom arises [9, 10]. Both categories were compared in a series of previous studies, and diverse conclusions came out [16–18].

However, according to the principle of personalized cancer medicine (PCM), there is no clear distinction among different disease conditions in the strategies mentioned above. The five-year survival rate for stage I patients after radical resection was over 80% and greatly decreased to 10% for stage IV [29]. And in the current study, the two-year DSS of stage IA was 91.4% and that of stage IIIC was only 28.3% (Figure 2(a)). In addition, there was a monotonous increasing of mortality risk with staging (Figure 2(c)). According to these disparities, it might result in deviation of prognosis monitoring if patients are being followed up equally and economic and psychological burden might also increase among patients. Although it has said that patients undergoing endoscopy have no need for routine surveillance unless they are symptomatic in the NCCN guideline, we still considered that it could be more personalized for various conditions.

In most of the studies, OS was applied to evaluate prognostic factors, such as TNM stage, high-risk factors, or other multivariable nomograms [23, 30, 31]. However, OS refers to the alive period from treatment to death, including those who died from cardiovascular disease, respiratory disease, or others. These might result in deviations. Moreover, considering the changing mortality risk with increased survival time, CS was a better choice to evaluate real-time prognosis. From a multicenter study, the application of CS not only contributes to clinical decision making by clinicians but also has health economic, social, and psychological benefits [22].

In the current study, we selected 9191 gastric cancer patients after gastrectomy from the SEER database. HR curves of stages I and IIA were flatter than those of stages IIB and III, and the latter group also had an obvious peak (Figure 2(b)). Then we used CS to evaluate surveillance strategies mainly according to the NCCN and JGCA guideline. As illustrated in Figures 3(a) and 3(b), there was no obvious difference between 3mCS and 6mCS or 6mCS and 12mCS and all were no less than 94% in stage I. It could be concluded that the interval of every follow-up could be prolonged to six months in the first three years and 12 months in the latter two years or much longer in stage I.

And as illustrated in Figure 2(b), the HR curve of stage IIA was flat, while there was a visible peak in stage IIB and an obvious rise compared to the former. Additionally, 3mCS and 6mCS or 6mCS and 12mCS of stage IIA showed smaller gaps than those in stage IIB (Figures 3(c) and 3(d)). Therefore, we considered that the trend of CS in stage IIA was more likely the same as that in stage I, while that in stage IIB was similar to that in stage III. And stage III patients had visible differences among CS from diverse intervals, and we considered that these patients should be followed up more frequently (Figures 3(e) and 3(f)).

From the above conclusions, we could further summarize that personalized surveillance strategy had its rationality and inevitability for the heterogeneity of patients' conditions. And stage I plus IIA patients could not be followed up so close, such as every six months or longer in the first period and annually thereafter. But being more frequently followed up, such as every three months or more intensively, for stage III plus stage IIB would be conducive for timely detection and treatment. Moreover, unnecessary frequent follow-up for early stage patients must imply high cost for detection such as blood test including tumor markers, whole abdomen computed tomography (CT), and ultrasound. And like a chain reaction, it must also result in psychology burden and improve the possibility of anxiety or other problems [20, 21].

Certainly, the current study had several limitations to be presented. Firstly, our analyses were based on retrospective data and selection principle was based on diagnosis, demographic and pathological characteristics, and other information existing in the SEER database. It might result in deviations on account of various diagnoses, treatment principles, and data quality among multiple medical centers. Secondly, many previous studies indicated that perioperative treatment was an important prognosis factor [32, 33]. It would be more rationalized and personalized if we could include perioperative treatment data, which is not detailed enough in the SEER database. Moreover, there was also no information about detailed treatment of each gastric cancer patient in the SEER database, such as surgical retreatment, palliative therapy, and relapse situation, which may lead to certain deviation. And the cohort was all from the SEER database and the conclusions were mostly suitable for Americans. Further analysis is required for Chinese patients.

## 5. Conclusions

In conclusion, we qualitatively described the defects of the surveillance carried out presently and put forward several simple ideas to improve. The present surveillance strategies have no detailed personalized solutions and are deviated from PCM. The current study used DSS, CS, and HR curve to describe survival conditions after gastrectomy. There is no need for early-stage patients to be followed up as frequent as locally advanced-stage patients. Stage I and IIA patients could be followed up every six months or longer in the first period (stage I was the first three years and stage IIB was the first two years according to the guideline) and annually thereafter, while stage IIB and III patients could be followed up every three months or more frequently. From the conclusions above, the rationality of monitoring, the timely treatment, health economics, and patient mentality will be improved for those with favorable conditions. Furthermore, we intended to collect more detailed data from multicenter databases and use appropriate mathematical models to conclude more reasonable follow-up intervals and it must require the efforts of all experts.

## Figures and Tables

**Table 1:** Demographic and pathological characteristics of patients from the SEER database.

Characteristic	All patients (no. = 9191)
	Mean or no.
Sex	
Male	5228 (56.9%)
Female	3963 (43.1%)
Age in years	
≤60	2790 (30.4%)
>60	6401 (69.6%)
Median (IQR)	69 (57-77)
Mean ± SD	66.6 ± 13.3
Race	
White	5319 (57.9%)
Black	1454 (15.8%)
Others^#^	2402 (26.1%)
Unknown	16 (0.2%)
Grade	
Well differentiated	326 (3.5%)
Moderately differentiated	2162 (23.5%)
Poorly differentiated	6185 (67.3%)
Undifferentiated	235 (2.6%)
Unknown	283 (3.1%)
Primary site	
Fundus	384 (4.2%)
Body	1022 (11.1%)
Antrum	3265 (35.5%)
Pylorus	569 (6.2%)
Lesser curvature	1603 (17.4%)
Greater curvature	648 (7.1%)
Overlapping lesion	808 (8.8%)
NOS	892 (9.7%)
Size	
≤4.5 cm	4737 (51.5%)
>4.5 cm	4454 (48.5%)
Mean ± SD (cm)	5.2 ± 3.9
T stage	
T1a	612 (6.6%)
T1b	1099 (12.0%)
T2	1127 (12.3%)
T3	3650 (39.7%)
T4a	1796 (19.5%)
T4b	907 (9.9%)
N stage	
N0	3361 (36.6%)
N1	1735 (18.9%)
N2	1798 (19.6%)
N3a	1659 (18.0%)
N3b	638 (6.9%)
TNM stage	
IA	1331 (14.5%)
IB	806 (8.8%)
IIA	1357 (14.8%)
IIB	1269 (13.7%)
IIIA	1287 (14.0%)
IIIB	1836 (20.0%)
IIIC	1305 (14.2%)
Number of LNs examined	
≤15	5491 (59.7%)
>15	3700 (40.3%)
Median (IQR)	13 (7-21)
Mean ± SD	15.5 ± 11.9
Number of positive LNs	
Median (IQR)	2 (0-6)
Mean ± SD	4.5 ± 6.7

No.: number of patients; SD: standard deviation; IQR: interquartile range; LNs: lymph nodes. ^#^American Indian/AK native or Asian/Pacific Islander.

**Table 2:** Univariate and multivariate Cox hazards regression analyses of independent prognosis factors.

	Univariate analysis		*P* value	Multivariate analysis		*P* value
	HR	95% CI		HR	95% CI	
Race						
White	Ref					
Black	0.981	0.912-1.0154	0.599			
Other^#^	0.684	0.640-0.730	<0.001			
Unknown	0.722	0.375-1.389	0.329			
Sex						
Male	Ref					
Female	0.967	0.917-1.020	0.217			
Age						
≤60	Ref					
>60	1.414	1.341-1.490	<0.001	1.724	1.621-1.833	<0.001
Grade						
Well differentiated	Ref					
Moderately differentiated	1.688	1.400-2.035	<0.001	1.083	0.895-1.308	0.409
Poorly differentiated	2.299	1.918-2.755	<0.001	1.254	1.044-1.508	0.016
Undifferentiated	2.303	1.814-2.924	<0.001	1.202	0.944-1.530	0.135
Unknown	1.633	1.286-2.074	<0.001	1.062	0.835-1.351	0.625
Tumor size						
≤.5 cm	Ref					
>4.5 cm	1.836	1.741-1.936	<0.001	1.097	1.037-1.160	0.001
TNM stage						
IA	Ref					
IB	2.005	1.691-2.377	<0.001	1.918	1.617-2.276	<0.001
IIA	3.039	2.627-3.516	<0.001	2.908	2.507-3.374	<0.001
IIB	4.942	4.290-5.693	<0.001	4,660	4.031-5.386	<0.001
IIIA	6.613	5.754-7.602	<0.001	6.455	5.592-7.451	<0.001
IIIB	8.414	7.357-9.624	<0.001	8.846	7.685-10.183	<0.001
IIIC	10.985	9.572-12.607	<0.001	11.944	10.324-13.819	<0.001
Number of LNs examined						
≤15	Ref					
>15	0.863	0.818-0.911	<0.01	0.664	0.628-0.702	<0.001

CI: confidence interval; HR: hazard risk; Ref: reference category; LNs: lymph nodes. ^#^American Indian/AK native or Asian/Pacific Islander.

## Data Availability

The data used to support the findings of this study are available from the corresponding author upon request.
